# Upregulated TCRζ improves cytokine secretion in T cells from patients with AML

**DOI:** 10.1186/s13045-015-0170-0

**Published:** 2015-06-18

**Authors:** Shaohua Chen, Xianfeng Zha, Li Shi, Lingling Zhou, Lijian Yang, Bo Li, Xiuli Wu, Jun Zhong, Tao Zhang, Yuhong Lu, Kanger Zhu, Yangqiu Li

**Affiliations:** Institute of Hematology, Medical College, Jinan University, Guangzhou, 510632 China; Department of Clinical Laboratory, First Affiliated Hospital, Jinan University, Guangzhou, 510632 China; Department of Hematology, First Affiliated Hospital, Jinan University, Guangzhou, 510632 China; Key Laboratory for Regenerative Medicine of Ministry of Education, Jinan University, Guangzhou, 510632 China

**Keywords:** Acute myeloid leukemia, T cells, TCRζ, Cytokine, Chemokine

## Abstract

Previous studies indicated that upregulating *TCR*ζ partially recovers T cell function in patients with leukemia. In this study, we characterized the cytokine profile of *TCR*ζ-transfected T cells from acute myeloid leukemia (AML) patients by Quantibody®Array Glass Chip. Firstly, the significantly lower expression of TCRζ in CD3^+^/TCRζ^+^ cells from AML patients was found. Increased secretion of IL-2, IL-8, IL-10, IL-13, IFN-γ, TNF-α, GM-CSF, growth-regulated oncogene (GRO), MIP-1b, and regulated on activation, normal T cell expressed and secreted (RANTES) could be detected in T cells from AML patients after TCRζ upregulating. We concluded that upregulating TCRζ in T cells from AML can alter the secretion profile of cytokines and chemokine which are involved in T cell proliferation and activation.

**Electronic supplementary material:**

The online version of this article (doi:10.1186/s13045-015-0170-0) contains supplementary material, which is available to authorized users.

## Findings

Acute myeloid leukemia (AML) is an aggressive disease with an unfavorable prognosis [1–3]. T cell immunodeficiency is a common characteristic in hematological malignancies which may be due to defective TCRζ. Previous studies showed that *TCR*ζupregulation could be induced in CD3^+^T cells from AML patients by IL-2, IL-7, and IL-12 [4]. In this study, we characterized the secretion profile of cytokines and chemokines related to T cell activation in TCRζ-IRES2-EGFP-transfected T cells from AML patients after TCRζupregulation.

**Fig. 1:**
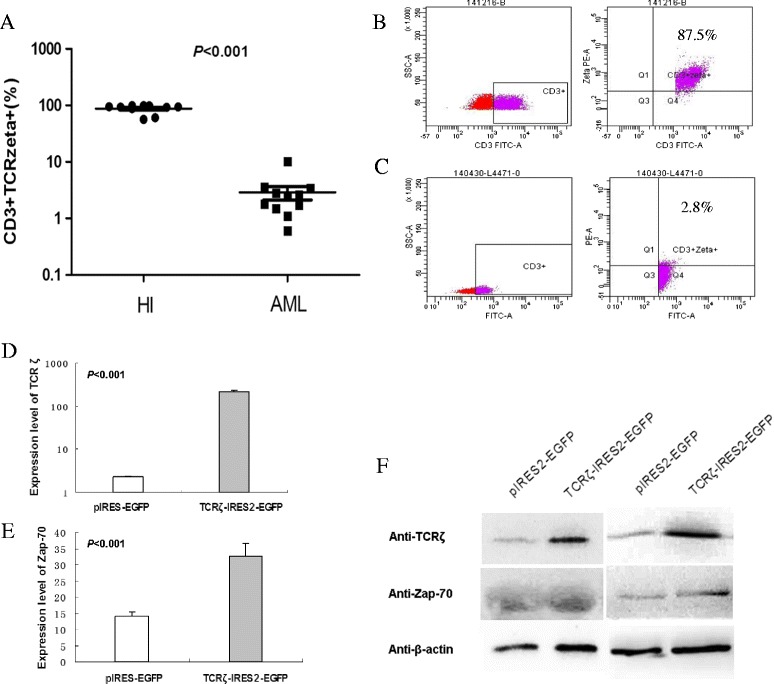
Expression of CD3^+^/TCRζ^+^ cells in PBMCs and expression of TCRζ and Zap-70 in TCRζ-transfected CD3^+^ T cells from patients with AML. **a** MFI of CD3^+^/TCRζ^+^ in PBMCs from AML patients and healthy individuals (*HI*) (*n* =10). **b** Percentage of CD3^+^/TCRζ^+^ cells in PBMCs from a healthy individual. **c** Percentage of CD3^+^/TCRζ^+^ in PBMCs from a patient with AML. **d** TCRζ gene expression levels. **e** Zap-70 gene expression level in TCRζ-transfected CD3^+^ T cells and control cells. **f** TCRζ and Zap-70 protein expression in transfected CD3^+^ T cells from two AML samples and control cells

First, significantly lower TCRζ expression in CD3^+^/TCRζ^+^ cells in AML (2.89 ± 2.6 %, *n* = 10) was found in comparison with healthy individuals (87.38 ± 15.67 %, *n* = 10) (*p* < 0.001) (Fig. 1a–c). This result further supported our previous finding that T cell immunodeficiency might be due to low TCRζ signaling in T cells [5–8].

CD3^+^T cells were sorted from PBMCs from four AML patients (Additional file 1: Table S1) who had TCRζ deficiency and then transfected with TCRζ-IRES2-EGFP or IRES2-EGFP, respectively, by nucleofection [9]. Significant upregulation of TCRζ in TCRζ-IRES2-EGFP-transfected CD3^+^T cells was confirmed. Similar results were found in *TCR*ζ downstream target factor Zap-70 (Fig. 1d–f). Thus, *TCR*ζ gene transfection could directly upregulate TCRζ and Zap-70 in T cells from AML patients as previously found in CML [9].

**Fig. 2:**
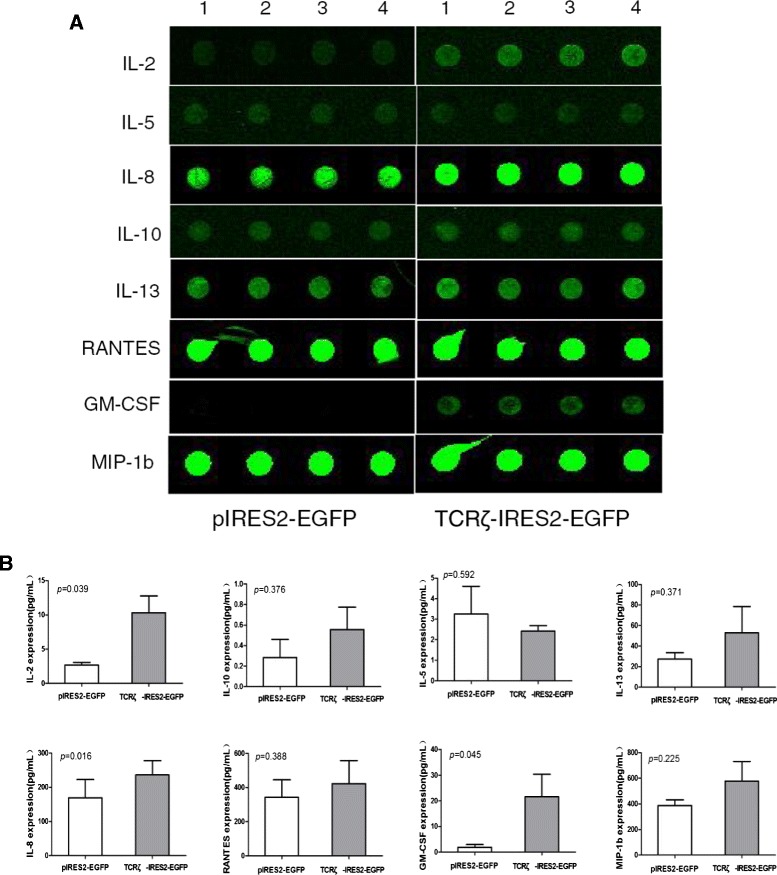
Detection of the IL-2, IL-5, IL-8, IL-10, IL-13, RANTES, GM-CSF, MIP-1b, IFN-γ, and TNF-α level secreted from T cells from AML patients using Quantibody® array. **a** Fluorescence intensity (concentration) from laser scanner results. *1–4*: four parallel wells for each sample. **b** The level of IL-2, IL-5, IL-8, IL-10, IL-13, RANTES, GM-CSF, and MIP-1b secreted from T cells from four cases with AML

Forced TCRζ chain expression can reverse TCR/CD3-mediated signaling abnormalities and defective IL-2 production in T cells [9, 10]. In this study, we used Quantibody®Array Glass Chip (www.raybiotech.com) to quantitatively measure 20 human cytokines and chemokines in supernatants from TCRζ-IRES2-EGFP-transfected and IRES2-EGFP-transfected T cells from AML patients (Additional file 2). Increased secretion of IL-2, IL-8, IL-10, IL-13, IFN-γ, TNF-α, GM-CSF, growth-regulated oncogene (GRO), MIP-1b, and regulated on activation, normal T cell expressed and secreted (RANTES) and decreased secretion of IL-5 were found, while the secretion level of IL-1α, IL-1β, IL-4, IL-6, and IL-12 had no obvious change after TCRζupregulation. Moreover, the changes in the secretion levels of IL-10, MCP-1, MIP-1a, MMP-1, and VEGF were different in different AML samples (Fig. 2). After TCRζ transfection, the IFN-γ secretion level was increased in all samples in the TCRζ-IRES2-EGFP group (median 71.46 pg/mL) compared with the pIRES2-EGFP group (median 42 pg/mL) (*P* = 0.253) because the basal level of IFN-γ in T cells from different AML patients was relatively different, ranging from 18.89 to 169.41 pg/mL in control cells and from 54.02 to 335.33 pg/mL in TCRζ-IRES2-EGFP cells. Thus, it could be understood that the increased secretion of IFN-γ was not statistically different in this study even though there was an obvious change in its level. Similar characteristic was found in TNF-α secretion level (Additional file 3: Figure S1). Interestingly, we found that the level of GM-CSF was significantly increased (21.63 ± 15.19 pg/mL for TCRζ-IRES2-EGFP cells vs. 1.96 ± 1.83 pg/mL for pIRES2-EGFP cells) (*p* = 0.045) (Fig. 2), and IL-13, which is secreted by activated T cells and has synergistic effects with GM-CSF and G-CSF, was also upregulated after TCRζ gene transfection (Fig. 2). Recently, increasing data have shown that GM-CSF has a variety of effects on the immune system including the activation of T cells, maturation of dendritic cells, and the ability to promote humoral and cell-mediated responses; thus, it has been incorporated into immunotherapy strategies [11, 12].

In conclusion, we characterized the profile of cytokines and chemokines secretion in T cells after TCRζ gene transfection. Most cytokines related to T cell proliferation and activation, such as IL-2, IFN-γ, and TNF-α, had increased secretion after TCRζ upregulating. Moreover, some of the Th1-associated CC subfamily chemokines, such as CCL4 and CCL5, may contribute to T cell activation via TCRζ upregulation. These results may further support the idea of the effects of upregulating TCRζ in T cell immunity.

## Additional files

Additional file 1: Table S1. Clinical data of AML patients. Additional file 2:
**Materials and methods.**
Additional file 3: Figure S1. Detection of the IFN-γ and TNF-α level in T cells from AML patients using Quantibody® array. A: Laser scanner fluorescence intensity (concentration) results. 1–4: samples from four cases with AML. B: The secretion level of IFN-γ and TNF-α in T cells from four cases with AML.
