# Supplementary material for: Upregulated TCRζ improves cytokine secretion in T cells from patients with AML
Source: J Hematol Oncol. 2015 Jun 18;8:72. doi: 10.1186/s13045-015-0170-0 (PMC4488036; doi:10.1186/s13045-015-0170-0)
Supplement: Additional file 1: Table S1. — Clinical data of AML patients. [file 13045_2015_170_MOESM1_ESM.doc]

Table 1 Clinical data of AML patients

| No. | Subtype | Sex | Age | WBC  (×10^9^/L) | Blast+promyelocyte cells (%) | Platelets  (×10^9^/L) |  |
| --- | --- | --- | --- | --- | --- | --- | --- |
| 1 | M5 | F | 49 | 163 | 64 | 35 |  |
| 2 | M4 | M | 50 | 421.1 | 84 | 40 |  |
| 3 | M5 | F | 63 | 247 | 98 | 25 |  |
| 4 | M4 | M | 77 | 164 | 61 | 18 |  |
