# Supplementary material for: Upregulated TCRζ improves cytokine secretion in T cells from patients with AML
Source: J Hematol Oncol. 2015 Jun 18;8:72. doi: 10.1186/s13045-015-0170-0 (PMC4488036; doi:10.1186/s13045-015-0170-0)
Supplement: Additional file 2: — Materials and methods. [file 13045_2015_170_MOESM2_ESM.doc]

**Materials and Methods**

**Samples**

The samples used in this study were obtained from 14 newly diagnosed, untreated (de novo) patients with AML including 6 males and 8 females (14-77 years old, median age: 49) who were classified according to morphology based on criteria defined by FAB classification. Among these samples, 4 were selected for the *TCRζ* gene transfection experiment. The clinical data of the patients are listed in Table 1. All of the procedures were conducted according to the guidelines of the Medical Ethics Committees of the Health Bureau of the Guangdong Province of China, and ethical approval was obtained from the Ethics Committee of Medical School of Jinan University for this study.

**human CD3+ T cell isolation and culture**

peripheral blood mononuclear cells (PBMCs) were isolated from heparinized venous blood by Ficoll-Paque gradient centrifugation. All procedures were conducted according to the guidelines of the Medical Ethics Committee of the Health Bureau of the Guangdong Province of China. The cells were collected, washed twice in Hank’s balanced salt solution, and re-suspended at a final concentration of 2 × 10^6^ cells/mL in complete RPMI 1640 medium (Invitrogen, Grand Island, NY) supplemented with 10% heat-inactivated fetal calf serum (FCS; HyClone, Logan, UT), 100 U/mL penicil**li**n, 100 µg/mL streptomycin, 2 mM L-glutamine, and 50 µM 2-mercaptoethanol. CD3+T cells were positively purified from freshly isolated PBMCs from four AML cases (Table 1) using CD3+ microbeads (Miltenyi Biotec, Bergisch Gladbach, Germany) according to the manufacturer’s protocol. Peripheral blood from 10 healthy individuals served as control.

**CD3+/TCRζ+ T cell expression**

CD3+/TCRζ+ T cell expression was measured in permeabilized cells by FCM using a fluorescein isothiocyanate (FITC)-conjugated mouse anti-human CD3 (OKT3) mAb (BioLegend, USA) and a phycoerythrin (PE)-conjugated mouse anti-human TCRζ (6B10.2) mAb (Santa Cruz Biotechnology, USA). For each sample, 10,000 events were acquired, and all cells were included in the forward scatter/side scatter (FwSc/SSc) gate. The TCRζ cell surface density was expressed as the average fluorescence intensity of the analyzed cell populations (mean fluorescence intensity, MFI). The data were processed using FlowJo software (Tree Star Inc., USA) [1].

**Construction of the TCRζ-pIRES2 -EGFP recombinant plasmid**

The plasmid used in this study is based on the pIRES2-EGFP vector (Clontech Laboratories, Inc., USA), which encodes an internal ribosome entry site followed by EGFP (enhanced green fluorescent protein). The sequence encoding the full-length *TCRζ* gene was amplified from cDNA that was prepared from a healthy individual, and it was cloned between the Nhe I and EcoRI restriction sites [1].

**TCRζ gene transduction in T cells**

CD3+ cells (1×10^7^) sorted from AML patients were resuspended in 0.1 mL of supplemented **Nucleofector** solution (human T cell **Nucleofector™** kit, Amaxa, Gaithersburg, MD) at room temperature. TCRζ-IRES2-EGFP and IRES2-EGFP (5 µg) were mixed with 0.1 mL of cell suspension, transferred to a 2.0 mm electroporation cuvette, and nucleofected using the Amaxa **Nucleofector** II apparatus according to the manufacturer’s guidelines. Storage of the cell suspension in human T cell **Nucleofector** solution for longer than 20 min was avoided because this reduces the cell viability and gene transfer efficiency. The cells were treated using the U-014 program. The transfected T cells were immediately transferred to pre-warmed complete culture medium and cultured in 12-well plates in a humidified incubator at 37°C and 5% CO_2_. The transfection efficiency was estimated in each experiment by scoring the number of double-positive CD3+ and GFP cells stained with a PE-conjugated mouse-anti-human CD3 antibody (eBioscience, USA) 18 h post transfection using FCM analysis [1-2].

**Real-time quantitative reverse transcription-polymerase chain reaction (qRT-PCR)**

The expression level of the *TCRζ* and *Zap-70* genes in cDNA from TCRζ-transfected CD3^+^ T cells and control cells was examined by SYBR Green I real-time PCR as previously described [3-5,2].

**Quantibody®Array Glass Chip analysis**

Cell supernatants were collected from TCRζ-IRES2-EGFP- or IRES2-EGFP-transfected CD3+ T cells 24 h after transfection. The cytokines were detected using Quantibody®Array (a quantitative array platform using multiplexed sandwich ELISA-based technology) according to the manufacturer’s protocol (RayBiotech, Norcross, GA, USA). The chip was used to detect 20 cytokines and chemokines including IL-1α, IL-1β, IL-2, IL-4, IL-5, IL-6, IL-8 (CXCL8), IL-10, IL-12, IL-13, interferon-γ (IFN-γ), tumor necrosis factor-α (TNF-α), granulocyte macrophage colony-stimulating factor (GM-CSF), growth-regulated oncogene (GRO, CXCL1), monocyte chemoattractant protein (MCP)-1 (CCL2), macrophage inflammatory protein (MIP)-1a (CCL3), MIP-1b (CCL4), matrix metal proteinase-1 (MMP-1), vascular endothelial growth factor (VEGP) and regulated upon activation normal T cell express secreted (RANTES, CCL5). Data extraction was performed by quantitative data analysis using Quantibody Q-Analyzer software [6].

**Western blot analysis**

Cd3+ t cells (1 × 10^6^ /0.2 mL) were stimulated with 10 µg/mL OKT3 for 2 min at 37°C 18 h after transfection. The reaction was stopped by the addition of 1 ml ice-cold stop buffer (PBS, 5 mM EDTA, 10 mM NaF, 10 mM sodium pyrophosphate, and 0.4 mM sodium vanadate), and the cell pellets were solubilized in RIPA lysis buffer (1×PBS, 1% Nonidet P-40, 0.5% sodium deoxycholate, 0.1% sodium dodecyl sulfate, 10 mmol/L phenylmethylsulfonyl fluoride, 1 µg/mL aprotinin, and 100 mmol/L sodium orthovanadate) and incubated on ice for 30 min to isolate total protein. Proteins (20 µg) were separated in a 12% SDS-PAGE gel and transferred to a PVDF membrane (Pall, USA) using a damp-dry transfer device (Bio-Rad, USA). After blocking for 1 h in 5% defatted milk powder dissolved in PBS, the membrane was washed and probed with mouse anti-β-actin (1:2,000, ProteinTech), rabbit anti-human TCRζ or anti-human Zap-70 (1:1,000, Cell Signaling Tech). The antibodies were detected with 1:10,000 horseradish peroxidase-conjugated goat anti-rabbit IgG and horseradish peroxidase- conjugated donkey anti-mouse IgG (Jackson ImmunoResearch). A western blotting luminol reagent (Lianke) was used to visualize bands corresponding to each antibody [27]. Blot images were captured using an Alliance 4.7 Gel doc system (UVI), and densitometric analysis of the bands was performed using Image J software (http://rsb.info.nih.gov/ij) [1].

**Statistical analysis**

Statistical analyses were performed using the independent samples t-test for the CD3+TCRζ+, *TCRζ* and *Zap*-70 expression levels, and the paired samples t-test was used for cytokines and chemokines and the TCRζ and Zap-70 protein levels in the TCRζ-IRES2-EGFP and IRES2-EGFP groups. Differences with a *p* < 0.05 were considered statistically significant.

**Reference**

1. Zha X, Chen S, Yang L, Shi L, Li B, Wu X, et al. Up-regulated TCRζ enhances interleukin-2 production in T-cells from patients with CML. [DNA Cell Biol](http://www.ncbi.nlm.nih.gov.ezp-prod1.hul.harvard.edu/pubmed/22873198). 2012; 31:1628-35.
2. Chen S, Huang X, Chen SH, Yang L, Shen Q, Zheng H, et al. The role of *BCL11B* in regulating the proliferation of human naïve T cells. Hum Immunol. 2012; 73:456-64.
3. Zha X, Yan X, Shen Q, Zhang Y, Wu X, Chen S, et al. Alternative expression of TCRζ related genes in the patients with chronic myeloid leukemia. J Hematol Oncol. 2012; 5:74.
4. Chen S, Yang L, Lu X, Li B, Chan JYH, Cai D, Li Y. Gene expression profiling of CD3γ, δ, ε and ζ chains in CD4+ and CD8+ T-cells from human umbilical cord blood. Hematology. 2010; 15:230-5.
5. Shen Q, Liu S, Chen Y, Yang L, Chen S, Wu X, et al. Proliferation inhibition and apoptosis induction of imatinib-resistant chronic myeloid leukemia cells via PPP2R5C down-regulation. J Hematol Oncol. 2013; 6:64.
6. Gao QT, Cheung JK, Li J, Jiang ZY, Chu GK, Duan R, et al. A Chinese herbal decoction, Danggui Buxue Tang, activates extracellular signal-regulated kinase in cultured T-lymphocytes. FEBS Letters. 2007; 581:5087-93.
