# Supplementary figures and images for: Upregulated TCRζ improves cytokine secretion in T cells from patients with AML
Source: J Hematol Oncol. 2015 Jun 18;8:72. doi: 10.1186/s13045-015-0170-0 (PMC4488036; doi:10.1186/s13045-015-0170-0)

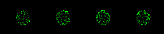


pIRES2-EGFP

A

B


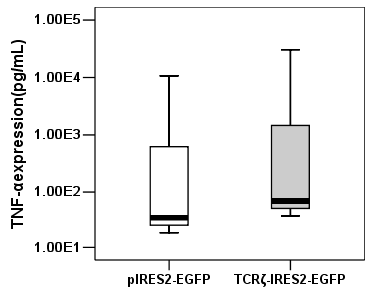


*P*=0.144


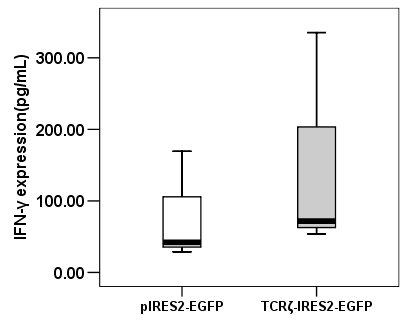


*P*=0.253

IFN-γ


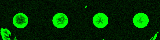

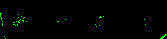

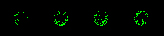

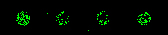

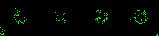

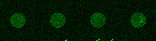

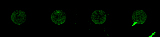

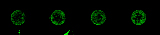


3

1

2

2

3

4

1

4

TNF-α


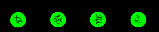

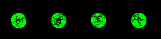

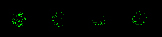

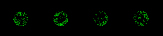

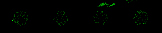

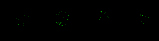

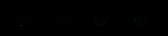


TCRζ-IRES2-EGFP

Supplement: Additional file 3: Figure S1. — Detection of the IFN-γ and TNF-α level in T cells from AML patients using Quantibody® array. A: Laser scanner fluorescence intensity (concentration) results. 1–4: samples from four cases with AML. B: The secretion level of IFN-γ and TNF-α in T cells from four cases with AML. [file 13045_2015_170_MOESM3_ESM.doc]
